# Male‐specific alterations in structure of isolation call sequences of mouse pups with 16p11.2 deletion

**DOI:** 10.1111/gbb.12681

**Published:** 2020-07-06

**Authors:** Swapna Agarwalla, Noelle S. Arroyo, Natalie E. Long, William T. O'Brien, Ted Abel, Sharba Bandyopadhyay

**Affiliations:** ^1^ Department of Electronics and Electrical Communication Engineering IIT Kharagpur Kharagpur India; ^2^ Department of Anesthesiology Weill Cornell Medicine New York New York USA; ^3^ Department of Biology University of Pennsylvania Philadelphia Pennsylvania USA; ^4^ Department of Pharmacology/ITMAT University of Pennsylvania, School of Medicine Philadelphia Pennsylvania USA; ^5^ Department of Molecular Physiology and Biophysics Iowa Neuroscience Institute, University of Iowa Iowa City Iowa USA; ^6^ Advanced Technology Development Centre (ATDC) IIT Kharagpur Kharagpur India

**Keywords:** 16p11.2 deletion, mutual information, pup isolation calls, USVs, vocalization sequence

## Abstract

16p11.2 deletion is one of the most common gene copy variations that increases the susceptibility to autism and other neurodevelopmental disorders. This syndrome leads to developmental delays, including speech impairment and delays in expressive language and communication skills. To study developmental impairment of vocal communication associated with 16p11.2 deletion syndrome, we used the 16p11.2del mouse model and performed an analysis of pup isolation calls (PICs). The earliest PICs at postnatal day 5 from 16p11.2del pups were found altered in a male‐specific fashion relative to wild‐type (WT) pups. Analysis of sequences of ultrasonic vocalizations (USVs) emitted by pups using mutual information between syllables at different positions in the USV spectrograms showed that dependencies exist between syllables in WT mice of both sexes. The order of syllables was not random; syllables were emitted in an ordered fashion. The structure observed in the WT pups was identified and the pattern of syllable sequences was considered typical for the mouse line. However, typical patterns were totally absent in the 16p11.2del male pups, showing on average random syllable sequences, while the 16p11.2del female pups had dependencies similar to the WT pups. Thus, we found that PICs were reduced in number in male 16p11.2 pups and their vocalizations lack the syllable sequence order emitted by WT males and females and 16p11.2 females. Therefore, our study is the first to reveal sex‐specific perinatal communication impairment in a mouse model of 16p11.2 deletion and applies a novel, more granular method of analysing the structure of USVs.

## Graphical abstract

Structure in sequence of syllables in mouse pup isolation calls is present in wild‐type pups but is absent in a male specific manner in pups of the 16p11.2del mice, a model of autism spectrum disorders and communication impairment.

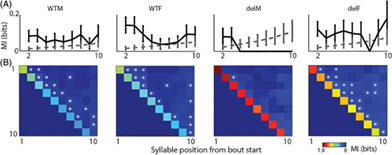

## 1 INTRODUCTION

Ultrasonic vocalizations (USVs) of mice, both in adults and in pups, are known to be of communicative significance.^1^, ^2^, ^3^, ^4^, ^5^, ^6^, ^7^, ^8^ Adult USVs have been widely studied and have shown that specific USVs are produced under different contexts, for example, in aversive or rewarding conditions.^8^, ^9^, ^10^, ^11^ Adult USVs have also been extensively studied in the context of courtship and mating behaviour.^5^, ^6^, ^8^, ^12^, ^13^, ^14^ Among mouse USVs, pup isolation calls (PICs) are particularly important, as they elicit searching and retrieval behaviour in the mother, and allow for individual recognition.^2^, ^3^, ^7^, ^15^ PICs are emitted when the pups are isolated from the nest or are cold, until postnatal day 13 (P13),^15^ although they are thought to start hearing at P10–11.^16^, ^17^ PICs, because of their communicative significance, provide a useful model to study developmental dysfunction in production of vocalization, especially in the context of autism spectrum disorders (ASDs) and related speech and language disability.^4^, ^18^, ^19^, ^20^, ^21^

Qualitative and quantitative analyses of USVs can help us in understanding various neurodevelopmental aspects like ethology, behavioural pharmacology, neurotoxicology, and behavioural neurogenetics. Various studies have reported changes in the acoustic features like call duration, peak frequency, bandwidth, peak amplitude and call rate with age in wild–type (WT)^22^, ^23^ as well as in ASD models.^24^, ^25^, ^26^ Furthermore, negative impact on mouse pup mother social communications was observed because of alteration in call sequences^27^ which is analogous to human studies in which participants felt more negative states on listening to crying episodes of ASD babies.^23^, ^28^ Grimsley et al.^23^ studied development of vocalizations in mice and, using Zipf's statistic^29^ and entropy analysis,^30^ showed that sequences of syllables produced by pups were non‐random. However, higher order structure in vocalizations or informative sequences that lead to structure has not been explored.

The main objective of this work is to identify the changes in properties of neonatal USVs and sequences of USVs in 16p11.2del compared with WT and if the changes are sex specific. We have investigated the acoustic features as well as the structure of the syllable sequences in steps of different orders starting from proportion of each syllable type to bouts of PICs. Using information theoretic analyses we determine the presence of informative components in the sequence of syllables for which we started with low order structure like transitions and then looked at high order structure, informative sequences. We find changes in syllable acoustic properties and syllable sequences that are specific to the male 16p11.2del pups. We specifically explore the possibility of presence of structure in PICs at the onset of vocalizations (P5) which corresponds to be in between the preterm (P3) and term (P7–P10) human infants.^31^ As there is great heterogeneity in ASD mouse models, we focussed on early communication calls and tried to find likely informative syllable sequences, which provide structure to PICs, using mutual information^30^ as a measure of dependence and transition probabilities between syllables.^32^, ^33^, ^34^ After obtaining specific structure in PIC sequences in the WT P5 pups, we characterized PICs in 16p11.2del mouse.^32^, ^34^, ^35^ PICs and we identified specific deficits in the male pups.

16p11.2 deletion syndrome, a disorder caused by deletion of ~27 genes on chromosome 16 at the p11.2 location, causes intellectual disability, developmental delay and many features of ASDs. Humans carrying the 16p11 deletion have impaired communication and socialization skills, as well as delayed development of speech and language.^19^, ^20^, ^21^, ^36^, ^37^, ^38^ Our findings of altered sequencing in mouse PICs strengthen the 16p11.2del mouse model and provide scope for further investigations to understand the circuit and molecular level manifestations of the disorder which lead to vocalization impairment associated with the disorder.

## 2 METHODS AND MATERIALS

All experiments were approved by the University of Pennsylvania Institutional Care and Use Committee and conducted in accordance with National Institute of Health guidelines. To generate experimental pups, B6129SF1/J (Jackson Lab # 101043) females were bred to B6129S‐Del(7Slx1b‐Sept1)4Aam/J (# 013128) males. Unless otherwise noted, all results were based on averaging data from within same sex and genotype pups within a litter, as opposed to analysing individual pups. We provide a rationale for grouping through clustering analysis presented at the end of the results section where variability within groups was addressed to study effects of litters within groups. Preliminary studies were performed on the 16p11.2del line to empirically determine that peak USV emissions occur around postnatal day 5, consistent with other reports.^15^, ^22^, ^23^, ^39^

### 2.1 Recording protocol

On postnatal day 5 (P5), male and female pups were individually placed on clean bedding material in a glass container (10 cm × 8 cm × 7 cm; open surface). The pup was placed in a Styrofoam container (20 cm × 21 cm × 12 cm) with a lid modified to hold the recording microphone inside of a sound‐attenuating cabinet (Med Associates, St. Albans, VT), for 5 min. USVs were recorded using an UltraSoundGate Condenser Microphone CM 16 (Avisoft Bioacoustics, Berlin, Germany), placed 5 cm above the pup. The signal was transduced with an UltraSoundGate 116H audio device (Avisoft Bioacoustics) and analysed with an Avisoft RECORDER software (version 4.2.14; Avisoft Bioacoustics). The signal was collected with a sampling rate of 375 kHz in a 16‐bit format. USVs were recorded during a 5‐min trial.

### 2.2 Pre‐processing

Each wav file was divided into 5 s epochs and read in MATLAB (Mathworks). To eliminate the effect of background noise, the signal was filtered by a Butterworth band pass filter of order 7, removing frequencies below 30 kHz and above 160 kHz.^24^ Furthermore, to capture important patterns in the signal leaving out low frequency noise, the signal was high pass filtered first by subtracting a 10 point moving average smoothed signal from the raw signal.

### 2.3 Segmentation of syllables

Short term Fourier transform (STFT) of each epoch was calculated using a Hamming window of length 1024 and an overlap of 75%. Syllables were identified by calculating the power concentrated in each frame divided by the power in all the frames. It was median filtered over 30 ms. Peaks are detected by using a peak detection, comparing each element of data to its neighbouring values. If an element of data was larger than both of its neighbours, the element was considered as a local peak. The local peaks were tracked until it went below a threshold (mean + 0.01 * STD). The process was continued iteratively until all the peaks are tracked and are stored as syllables.

### 2.4 Classification of syllables

**FIGURE 1:**
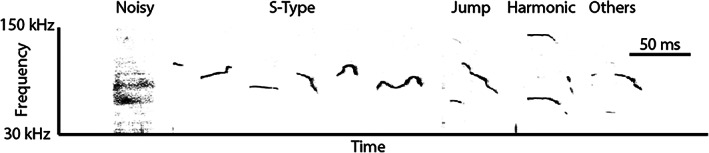
Example spectrograms of different types of syllables in PICs. Spectrograms of a total of 10 syllables are shown. The first syllable is of the noisy (N) type, the second through seventh syllables no pitch jump and are characterized as the S‐type. The next three spectrograms are respectively, jump (J) type (contains 1 pitch jump), harmonic (H) type and Others (O) type (containing 2 or more pitch jumps)

Pitch jump is a distinctive feature for classification of mouse vocalizations as shown by Holy and Guo.^14^ Our classification is based on the presence and absence of pitch jumps as done by Holy and Guo.^14^ First from the STFT the variance in frequency content was used to determine if the energy content was broadband, and classified as N‐type. Next harmonic content was checked based on spectral peaks. Syllables were classified as H‐type if significant peaks were present as harmonics of each other in at least three frames. For further classification, pitch jump was detected by calculating the change in gradient direction both frequency and time axis of the STFT using a Sobel operator. The pitch gradient magnitude was calculated. Peaks in the gradient were found whenever there were pitch jumps. The number of significant peaks is an indicator of the jumps in pitch. The threshold to determine peak in pitch gradient contours was decided blindly, without knowledge of genotype or sex. In the absence of pitch jumps syllables were either tonal with single spectral peak or S‐type; syllables with pitch jumps were either with single jump, J‐type or with multiple jumps and all other types were classified as Others or O‐type (Figure 1). There is a possibility of syllable misclassification, especially H types as S types in the delM and delF groups, because of any frequency components in the vocalizations above 160 kHz. Thus we repeated the classification with the upper cut‐off frequency as 180 kHz instead of 160 kHz (see in Section 2.2). There was absolutely no change in any of the syllable types with the two upper cut off frequencies. The distributions of syllable types for each group were exactly the same with 180 and 160 kHz (Figure S1). All subtypes of syllables were also present (Figure S2) as observed in other studies^5^, ^8^, ^9^, ^23^, ^40^ however, we used only pitch jump as the primary classification criteria based on Holy and Guo^14^ to restrict the number of broad classes enabling our analyses requiring large sample sizes. Furthermore, pitch jump based classification of syllables shown by Holy and Guo through isomaps^14^ is inherently tied to the vocalization production machinery.

### 2.5 Calculation of joint distributions and *MI* based dependence

Syllable to syllable *k*‐step transition probabilities *P*_*k*_(*S*_*i*_, *S*_*j*_), which are equivalent to elements of the joint probability distribution, were estimated from the data. Each element denotes the probability of observing the *j*‐type of syllable *k* steps after observing an *i*‐type syllable, where *i* and *j* vary from 1 to 5 (the 5 types of syllables observed). Lack of a particular combination leading to ‘0’ values in the joint distribution were corrected by the Krischevsky and Trofimov correction.^41^ Mutual information or *MI*, between two random variables *X* and *Y*, quantifies total dependence between the two random variables^30^ and can be computed from the joint distribution *P*(*X*, *Y*) and its marginal distributions *P*(*X*) and *P*(*Y*) as:

(1) $$\mathrm{MI}\left(X;Y\right)=\sum_{\mathrm{all}\;X}\sum_{\mathrm{all}\;Y}P\left(X=x,Y=y\right)\log_{2}\frac{P\left(X=x,Y=y\right)}{P\left(X=x\right)P\left(Y=y\right)}$$

By considering the syllables at a particular position as the random variable *X* and syllables after *k*‐steps as the random variable *Y*, which take on values *x* = 1–5 and *y* = 1–5 (the five possible values the random variables can take are the five possible syllable types) one can compute the dependence or *MI* between syllables in these two positions in a bout of syllables (Figure S3(A)–(C)). If there is no structure in the syllable sequences then the syllables should occur randomly and they would be independent of each other. If there is significant dependence between syllables at different positions, which means there is structure in the syllable sequences, that would be captured in non‐zero *MI*. The degree of dependence is given by the magnitude of *MI*, measured in units of bits. The above measurement is very sensitive to bias^42^, ^43^, ^44^, ^45^ and is only a raw estimate (*I*_raw_) and can lead to erroneous results because of limited data size in estimating the probability distributions. The above problem is circumvented in two ways, as described in the next section, through bootstrap removal of bias^46^ and comparing the *MI* estimates with scrambled syllable order in sequences of vocalizations to get only significant estimates of *MI*.

#### 2.5.1 Removal of bias in mutual information estimates and significance analysis

To remove bias in *MI* estimates, bootstrap debiasing was employed, which was based on a resampling technique that provides auxiliary information like bias and confidence interval.^46^ Each bootstrap dataset has the same number of elements as the original one. It was obtained by randomly selecting syllables keeping the transition intact between every two syllables in the original sequence (with replacement) and then mutual information is calculated. The sequence was bootstrapped 2000 times. A collection of estimates $\left\{I_{1}^{*},I_{2}^{*},I_{3}^{*},\ldots ,I_{N}^{*}\right\}$ of *MI* is obtained where *N* = 2000, and the mean of the set is called *I*_*BS*_. The debiased estimate of *MI* is given by 2 * *I*_raw_ − *I*_*BS*_.^45^ From the set of bootstrap estimates 95% confidence intervals were determined. We further compare the lower confidence interval with upper confidence interval of the estimate of ‘0’ *MI* obtained similarly as above but now by not keeping the transitions intact, that is by randomly scrambling the sequences which leads to the estimates of ‘0’ *MI* and its confidence interval from the same data set with same number of syllables and other statistics intact except the order of the syllables in a sequence (Figure S3(D)). When the confidence intervals of *MI* of the data and the scrambled data did not overlap, the estimated *MI* was considered to be significant. Thus we minimize the possibility (<5%) of spurious *MI* because of limited data size and variability.

### 2.6 Calculation of Kullback Leibler divergence between distributions

We quantified differences between distributions of different syllable types produced by the groups of pups using an information theoretic distance metric, Kullback Leibler divergence (*KLD*)^30^, ^47^ which makes no assumptions about the data. The same method is also applied to quantify differences in joint distributions of different syllable to syllable transition combinations. To compute *KLD* between the distributions *p* and *q* taking on values over the same set (in our case syllables produced by two different groups of pups, for example WTM (wild‐type male) and delM (16p11.2del male) taking values of different syllable types with probabilities *p*(*x*) and *q*(*x*), *x* being a syllable type, or the syllable to syllable transitions produced by the two groups) is computed as follows:

(2) $$\mathrm{KLD}\left(p\|q\right)=\sum_{\mathrm{all}\;X}p\left(x\right)\log_{2}\frac{p\left(x\right)}{q\left(x\right)}$$

As for *MI*, we performed debiasing of *KLD* using bootstrap resampling and consider significance in the same way with 95% confidence intervals.

## 3 RESULTS

The data presented in the study were collected from postnatal day 5 (P5) pups of four groups, namely WTM, WTF (wild‐type female), delM and delF (16p11.2del female). Isolation calls were recorded from 17 WTM, 13 WTF, 12 delM and 12 delF pups. The total number of syllables detected (Section 2) in 5 min in each group were as follows: WTM 7753 (mean per animal 456 ± 183), WTF 6143 (473 ± 227.0), delM 3649 (mean 304 ± 168) and delF 4683 (390 ± 221) and are significantly different (one way ANOVA, *p* < 0.001).

### 3.1 Types of syllables

Syllables observed in mouse vocalizations and PICs have been classified in a variety of ways, depending on the spectrotemporal features emphasized and characterized.^14^, ^23^, ^25^ Scattoni and colleagues^25^ used 10 categories, namely, complex, harmonic, upward, downward, chevron, 2 syllable, shorts, composite, frequency steps and flat (Figure S2). The above classification has been used by others with modification.^23^, ^48^ In the current work, the entire data set of detected syllables have been classified into five types of syllables (Figure 1) based on pitch jumps, a distinctive feature^14^ in USVs (Section 2). Using five classes of syllables based on pitch jumps also allows information theoretic calculations to be done reliably, as sufficient number of utterances of each kind need to be present. All types of syllables were found to be present in each of the groups of animals considered. Since all types of syllables were present in the delM and delF groups, the ability to produce the syllables is present in the 16p11.2del mouse pups, indicating that the syllable production machinery is intact and not fundamentally different in the different groups of animals. It is more likely that if any alterations are present, it is in the relative occurrence probabilities of syllables along with structure in sequences of syllables produced.

### 3.2 Basic call features

In order to elucidate possible alterations in PICs of the different groups we first quantified any possible differences in the basic call features like: call rate, call duration and mean peak frequency. Mean call rate of a pup for WTM, WTF, delM and delF were 91 ± 37, 95 ± 45, 61 ± 33 and 78 ± 44 calls per minute respectively. The mean call rate was significantly different among groups (one‐way ANOVA, *p* < 0.001). The delM group had lower call rate compared with WTM and WTF. Mean peak frequency for WTM, WTF, delM and delF were 69.3 ± 16, 68.7 ± 14, 75.5 ± 16 and 73 ± 16 kHz respectively(one‐way ANOVA, *p* < 0.001). Both the delM and delF pups showed significantly higher mean peak frequencies compared with the WTM and WTF groups. Mean call durations for WTM,WTF, delM and delF pups were 54 ± 22, 53 ± 20,40 ± 22 and 42 ± 20 ms respectively and were significantly different (one‐way ANOVA, *p* < 0.001). Both the delM and delF pups (40 ± 22 and 42 ± 20 ms) had significantly lower call durations. We also analysed differences between groups of pups in mean call duration of each syllable type and mean peak frequency of each syllable type. The results are summarized in Figure S8. There were no systematic differences based on each syllable type, which could be attributed to the delM or delF genotype. Thus the overall features independent of syllable type reflect correlational differences with genotype.

### 3.3 Probability of occurrence of different syllable types

**FIGURE 2:**
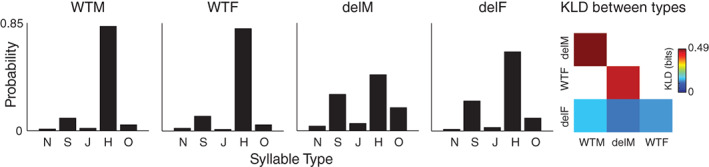
Probability of occurrence of each syllable type. The first four plots show the distributions of probability of occurrence of each of the five syllable types (N, S, J, H and O) in the different groups of pups identified in the title of each subplot. The plot to the right shows the *KLD* between the distributions to the left for all pairs of groups of pups. Asterisks indicate significance at 95% confidence

To further understand the differences in PICs across sexes and genotypes, we computed the distributions or probability of occurrence of the different syllable types. The four histograms in Figure 2 (left) show the probability of occurrence of each syllable type in the different groups of animals. The WTM and WTF distributions appeared similar and were robustly different from the delM and delF groups. The delM distribution appeared the most dissimilar from the rest. We quantify the differences in the distributions using *KLD* (Section 2) which is an information theoretic measure for distance between distributions. *KLD* is also susceptible to bias^45^, ^47^ and we present debiased significant values as done for *MI* (Section 2). KL distance in bits was calculated between all pairs of groups and the results are summarized in the matrix plot to the right in Figure 2. The WTM and WTF were not different from each other showing no sex based difference in distributions of syllable types in the WT. The delM distribution was the most different from the WTM and WTF groups with the largest *KLD*s. The delF distribution was also different from the rest but the *KLD*s are much smaller than those of the delM group. Thus a primarily male specific difference was observed in the 16p11.2del pups in the distribution of syllable types.

### 3.4 Bouts in PICs

**FIGURE 3:**
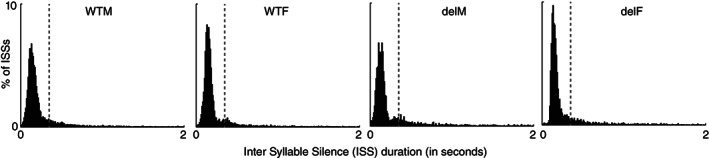
Inter syllable silence (ISS) distributions. The four distributions depict the inter‐syllable silences observed in each group of PICs. The vertical dashed line (at 350 ms) marks mean + 1 * STD of the overall data

Mouse USVs have bouts of calls separated by gaps of silences.^14^, ^23^, ^40^ The sequences of syllables emitted by each mouse pup have been divided into bouts by considering the distribution of silences between syllables (Figure S3(A)). The mean inter syllable silence (ISS) duration of all sequences was 200 ms with a standard deviation of 150 ms. When the silence interval between two successive syllables was greater than 350 ms (mean + STD), it was considered to be the end of the previous or start of a new bout (Figure S3(A)). Systematic variation in choice of the end of bout silence duration is considered later and shown to produce no variation in our main results and conclusions. The ISS distributions of each category of pups (up to 2 s) are shown (Figure 3), ignoring the time >2 s. The vertical dashed line in each subplot denotes 350 ms, the threshold for end of bout silence. To consider the effect of the threshold it was varied from mean + 1 * STD in steps of 0.5 STD up to 3 STD, that is 350–650 ms (Figures S4 and S5). Considering 350 ms as the threshold for starting of a new bout in a sequence of PICs, in WTM, WTF, delM and delF, the total bouts present were 1469 (mean 86 ± 21), 1070 (mean 82 ± 35), 866 (mean 72 ± 26) and 969 (mean 81 ± 30) respectively. The mean bout count was not significantly different in any of the cases (one way ANOVA, *p* = 0.05).

### 3.5 Information theoretic structure in sequences of syllables

**FIGURE 4:**
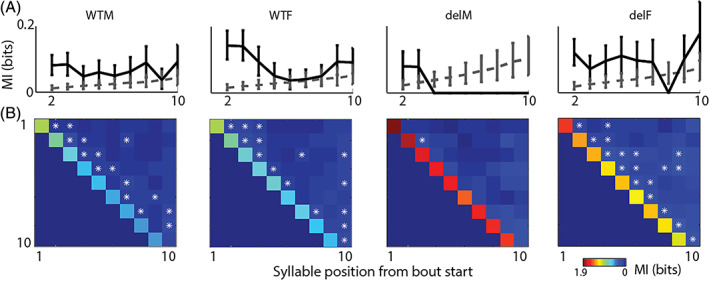
*MI* between the first and the *n*th syllable from the starting of a bout. The four plots show the dependence (quantified by *MI* in bits) between the first syllable and the subsequent syllables in the four groups WTM, WTF, delM and delF. The actual *MI* is plotted in thick black line with error bars showing 95% confidence intervals. The dashed thin gray line shows the *MI* (with 95% confidence intervals) between the syllables at the same positions when the syllable sequences in bouts were randomly scrambled (Section 2). (A) Non‐overlapping 95% confidence intervals indicate significant dependence with the first syllable of a bout. (B) The 4 matrices represent the *MI* calculated as in (A) with each row showing the *MI* for the *n*th syllable with the 1st (row 1), 2nd (row 2), 3rd (row 3) and so on. The diagonal elements show the entropy of the syllable in the corresponding position from the bout start. Asterisks mark significant *MI* (95% confidence)

Having quantified the basic differences in the individual syllables (and silences) of PICs in the WT and 16p11.2del male and female pups we then investigated higher order structure in the sequences of syllables. First, we asked if there was any dependence between syllables, that is, if there is any order in the occurrence of the syllables or if they occur randomly. To test the above, we compute *MI* (Section 2) between the syllable at the starting of a bout and the syllable at subsequent positions of a bout. Significant dependence or *MI* between the syllables treated as random variables would mean presence of structure in the sequence while lack of dependence or ‘0’ *MI* would indicate lack of structure. Figure 4(A) shows the debiased *MI* between the first syllable and the *n*th (*n* = 2, 3, …) syllable (thick line) with 95% confidence intervals for all the four groups. The thin dashed line in each case shows the *MI* with 95% confidence intervals for the scrambled sequences indicating the *MI* for no dependence. *MI* estimates with no overlap between the two confidence intervals at each position are significant. Thus in the WTM and WTF we see clear dependence or structure in the syllable sequences up to the fourth position from the starting of a bout. Such dependence is clearly absent in the delM population with no dependence between the starting syllable of a bout and any of the subsequent syllables. In the delF population dependence is present up to the third position. Hence the structure in sequences of syllables present in the WT PICs is completely absent in that of the delM pups. The above alteration in structure is primarily male specific as the sequences produced in delF population do have structure. Since the above analysis is dependent on starting of a bout, which is defined by the choice of threshold in Figure 3 we vary the threshold (Figure S4(A)) and do the same analysis considering larger silence durations to precede the start of bouts. The results shown for all four groups of pups in Figure S4(B), indicate that our conclusion above is independent of the criteria of marking the beginning of a bout of PICs. Making the silence duration marking the transition of bouts systematically longer does not change the observed degree or length of dependence between the bout starting syllable and subsequent syllables in any of the four groups.

To understand the higher order structure in the sequences, the above analysis was extended by computing *MI* between the second syllable in a bout and every successive syllable and further between the third and following syllables and so on. The results for each group are summarized in Figure 4(B) in the form of matrices with each row starting with the *MI* between the syllable at each position with itself (diagonal elements in the matrix) followed by *MI* with successive positions. The diagonal element is simply the entropy^23^, ^30^ in the *n*th position after bout start, and essentially quantifies the randomness of syllables at that particular position. As the bout gets longer, both in the WTM and WTF, and also in the delF, the entropy at each position from bout start decreases, making the subsequent syllables less random with length of the bout. Thus *MI* calculated between first, second or subsequent syllables with next syllables signifies how much of the uncertainty (entropy) is reduced by knowledge of a previous syllable. The reduction of uncertainty over the length of the bout varies between ~4 and ~30% in WTM, WTF and delF, while there is no significant reduction in uncertainty in case of delM. Given the observed dependence we next investigated the nature of syllable to syllable transitions in order to understand the cause of the presence of structure in the sequences.

### 3.6 Transition probabilities of two successive syllables

**FIGURE 5:**
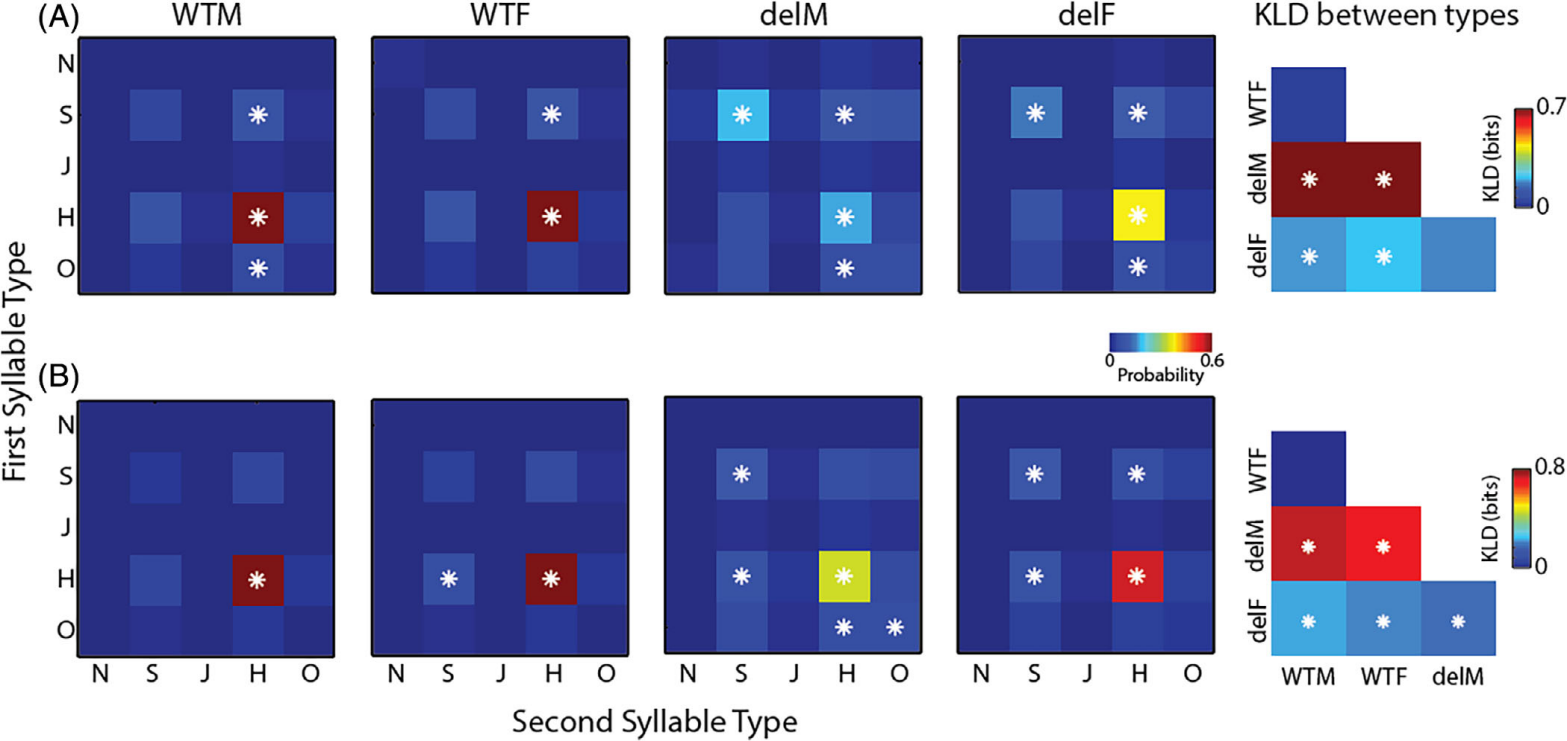
Syllable to syllable transition probability matrices. (A) Joint probability distributions of syllable type to syllable type transition (Figure S6) considering starting two syllables in bouts is depicted in each of first four matrices in the row for the four groups of pups. Asterisks indicate significance at 95% confidence. The lower diagonal matrix plot to the far right quantifies the *KLD* between joint distributions (left) of each pair of groups. (B) The bottom row is arranged in the same way as in (A) and shows the joint distributions for any successive two syllables (Figure S6) and the corresponding *KLD*s for each pair

In order to understand the nature of the structure, sequences of syllables were analysed considering two successive syllables – first considering only the starting two syllables in bouts and secondly considering any two successive syllables within bouts (Figure S6). Syllable to syllable transitions in both cases were quantified using the joint probability distributions; that is, the probability of occurrence of every possible pair of syllable types. The joint distributions are shown in the form of matrices in Figure 5(A) (first two syllables of a bout) and (B) (any successive two successive syllables) for all four groups of animals. Clearly in the WT groups the harmonic to harmonic transitions dominate with significant probabilities (based on 95% confidence intervals). S‐type to harmonic type was also common transitions. These types of transitions in the first two positions or in two successive syllables render structure to the sequences. Such clear transition types present in the WT is lost in the del‐groups particularly the delM group with the emergence of S‐type to S‐type transitions with equal probability as the harmonic to harmonic transitions in the delM group. The delF joint probability distributions also change but not to the degree of the delM group changes. We quantify the differences in the joint distributions again using *KLD* between every pair of groups. The *KLD* based quantification is summarized to the extreme right in Figure 5. Clearly the delM joint distributions were starkly different (95% confidence intervals) from the WTM and WTF, while the delF group is mildly different from the WT groups. The WT groups did not show any sex‐specific differences. Thus the male specific lack of dependence in sequences in the del‐group, at least for the first two positions can be attributed to the lack of high probability of a particular transition type (namely harmonic to harmonic) that is present in the WT groups and also somewhat in the delF group. Because the starting two syllables of a bout depends on the criteria or threshold silence duration marking the transition from one bout to another, we tested whether the joint distributions were different by changing the threshold duration of silence for bout end (Figure S3(A)). We found that the joint distributions for different threshold values (as in Figure S4) were all highly correlated with each other (Figure S5), indicating that our analysis did not depend on the choice of bout end silence duration as long as it was 1 * STD above the mean ISS.

### 3.7 Transition probabilities of three successive syllables

**FIGURE 6:**
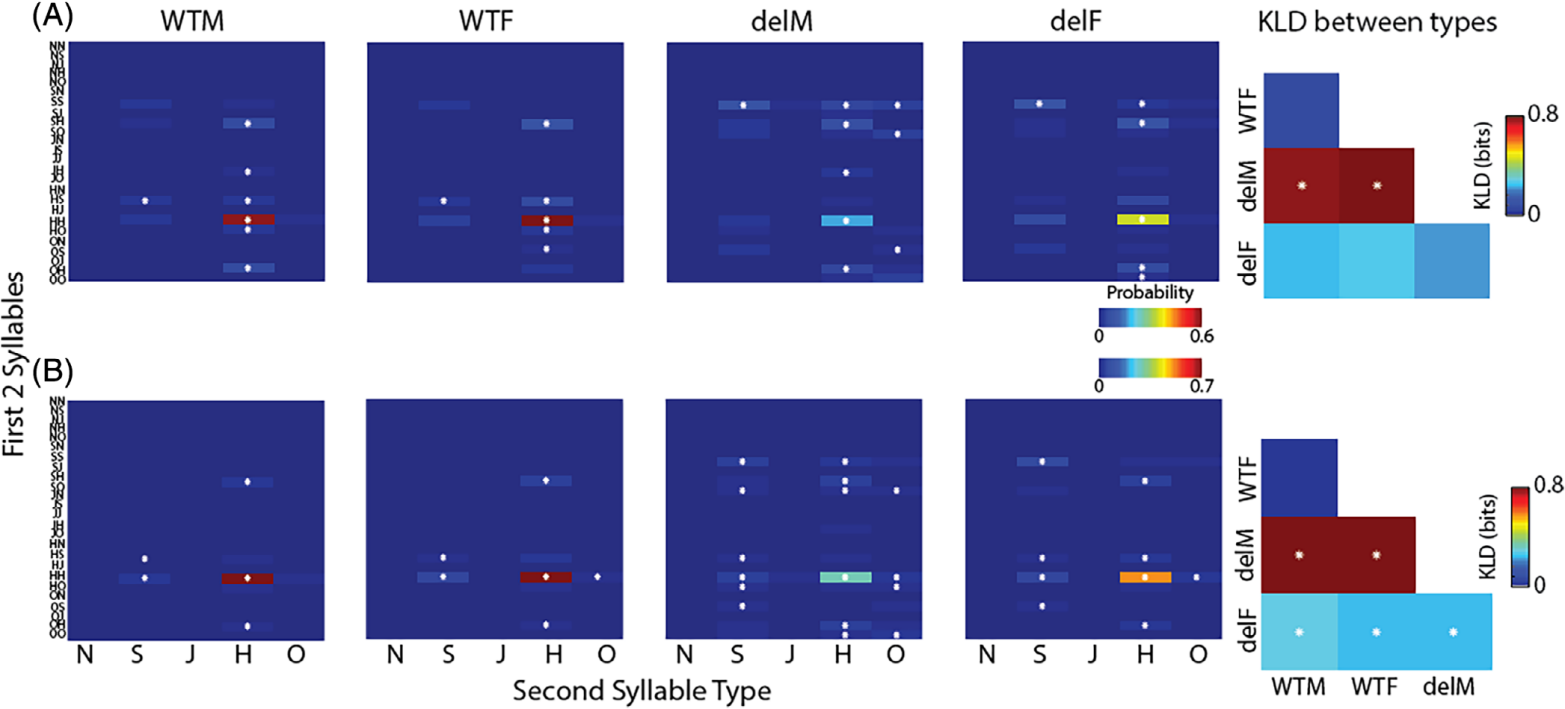
Transition probability matrices for three successive syllables. (A, B) are arranged identically to Figure 5(A), (B). In this case the joint distributions shown are for three successive syllables either from the beginning of bouts (A) or anywhere in bouts (B). The transition probability matrices have 125 elements with all possible pairs in the first two positions shown along the ordinate and the syllable type in the third position shown along the abscissae

We extend the analysis in the above section, of two successive syllables, to three successive syllables – both for the first three syllables of bouts and also any three successive syllables in a bout. Figure 6 summarizes the results and is arranged the same way as Figure 5 with the top row (Figure 6(A)) showing results of analysis of the first three syllables in a bout and the bottom row (Figure 6(B)) showing results of analysis of any three successive syllables in a bout. In this case the joint distribution consists of 125 possible pairs of transitions or triplets of syllable types, the 25 rows of the joint distribution matrices show the possible pairs of syllable types in the first two positions of the three syllables considered and the 5 columns depict the third syllable type. Clearly the WT groups have similar joint distributions with most of the probability mass being concentrated on harmonic type column and the harmonic to S‐type or harmonic to harmonic rows. The significant probability masses in the joint distributions in delM group are much more scattered in different types of possible transition pairs than that of the delF group. The quantification of differences between the joint distributions of every pair of groups, shown to the far right, reiterates the male specificity of sequence alteration. The results are similar to the observations of two successive syllables. Further, as for pairs of syllables, in this case also the WTM (WT Male) and WTF (WT Female) distributions were not significantly different.

Thus by considering syllable type to syllable type transitions in one step or two steps from beginning of a bout or otherwise, clear patterns are observed showing prevalence of particular types of syllables to occur in pairs or triplets in the WT population and also to a large extent in the delF(16p11.2del female) population. However, these patterns are not as clear in the delM (16p11.2del male) population particularly. Thus, the dependence between syllables observed (Figure 4) in WT groups and delF groups and its absence in the delM group can be attributed to the above. The analysis can be extended to successive four syllables and more but that would require much more data to accurately estimate the joint probabilities of each combination (their number increases exponentially) and is not possible to estimate with the current dataset.

### 3.8 High probability sequences produced in the different groups

With the limited data we therefore consider the types of sequences that are produced in the PICs of the different groups which are above chance. Given the probability of occurrence of each type of syllable (Figure 2) at any location in the bout we considered the types that occur above chance levels if the syllables in each position were drawn randomly from the overall probability distribution.

For each group of animals, we looked at the significance of occurrence of each syllable at different positions given the previous syllables. For the first position in a bout we considered the probability of occurrence of each syllable type in the beginning of the bout. The probability of each type to be in the first position of a bout is compared with the overall probability of occurrence of that syllable (Figure 2), that is, had the syllables been occurring randomly based on their respective occurrence probabilities. Syllables with higher (95% confidence) probability of occurrence than overall were considered as significant. The process was continued for each of the subsequent positions keeping the previous syllable types fixed until there were no significant syllables. In the above manner we find sequences that occur above chance and obtain the sequences that render structure to the PICs in different groups.

**FIGURE 7:**
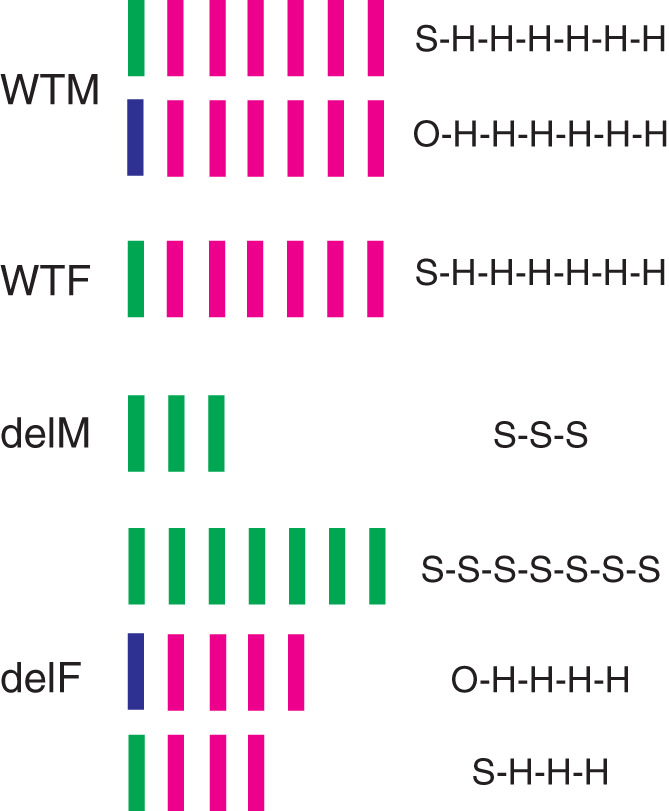
Significant syllable sequences of each group of pups. The most significant syllable sequences that were produced in each group of pups are represented. The WTM, WTF, delM and delF groups had 2, 1, 1 and 3 such sequences respectively. These consisted of three syllable types S‐type (green), harmonic type (magenta) and other (multiple pitch jumps, blue). The WTM and WTF shared one sequence while parts of the WT sequences were present in the delF group. The sequences with successive S‐types were exclusive to the del group of pups

Tracking sequences in the above manner from the start of bouts we found the significant sequences of syllables in each group summarized in Figure 7. The WTM and WTF produced two and one significant sequence respectively, of which the one in the WTF was also present in the WTM (S‐type followed by six consecutive harmonic types). The other significant sequence in the WTM was a sequence starting with ‘O' or Other‐type followed by six harmonic types. The delF group produced three significant sequences which were shorter versions of those observed in the WT groups and another that was a sequence of seven consecutive S‐type syllables. The delM group also produced one significant sequence that had only three consecutive S‐types, a shortened version of the third significant sequence in the delF group. From the sequences obtained, we also see the clear difference of the delM group from the rest. Again, the delF group had sequences similar to WT groups and also a sequence that is similar to the only sequence observed in the delM group.

### 3.9 Alterations in structure of syllables were not litter specific

Although we found clear male specific differences in the del‐group of mice in all our analyses there were some degree of similarities in the joint distributions. Furthermore, in the delF group we found more similarities with the WT group and some similarities with the delM group. In order to explain the similarities we asked whether the differences could be attributed to specific litters of mice in each group or whether any pup in a litter of each of the del groups could exhibit the differences. For these reasons we revisited our analyses of probability of occurrence of each syllable type and joint distributions of 1‐step and 2‐step syllable to syllable transitions and performed correlation of syllable (or transition) distribution based clustering of mouse pups.

**FIGURE 8:**
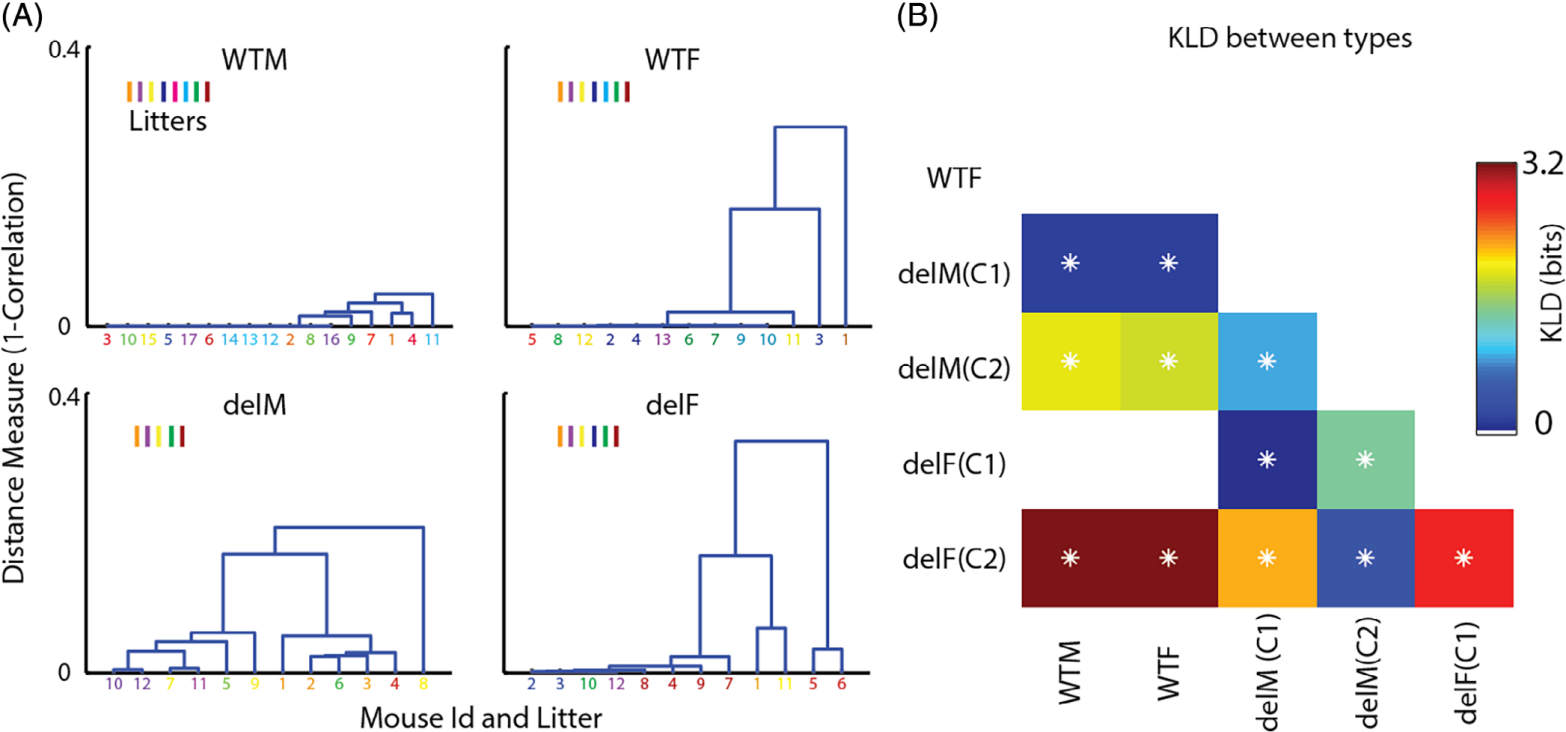
Clustering of pups based on syllable type distributions. (A) Dendrograms based on correlations (*ρ*) between distributions of syllable types of each pup were created by merging together minimally distant distribution (distance = 1 − *ρ*). The merges are depicted in the four dendrograms for each group of mice identified above every subplot. (B) Based on clusters of pups formed, *KLD* between distributions of each cluster/group pairs were computed as in Figure 2. Asterisks show significance at 95% confidence

We considered distance between distributions as 1 − *ρ* (*ρ* being the correlation between distributions) to cluster or group together the distributions with least distances.^49^ The above process allowed creation of dendrograms^49^ of groupings of distributions from each mouse. First such clustering was performed based on the distribution of probability of occurrence of each syllable type for each mouse. Figure 8(A) shows the obtained dendrograms in each group of pups with the mouse numbers are on the abscissae in colours depicting litter identity and the ordinate shows the distance (1 − *ρ*). The pups marked with same colour come from the same litter (WTM (8), WTF (7), delM (5) and delF (6), the number represent different litters). The dendrogram shows merging of the pup numbers into groups at varying degrees of distances. Clearly the WTM showed the least distances between pups and all of them could be merged into a single cluster. By using the maximum distance observed in the dendrograms in the WTM group as the threshold for grouping (distances above the threshold distance would mean different clusters) we grouped pups in the other categories. In WTF, out of 12 pups, 10 pups formed a single group and 2 were significantly different and were left out in further analyses of Figure 8. In the delM, five distinct clusters were obtained of which three clusters had one animal each (animal numbers 1, 8 and 9) and the other two clusters had five (animal numbers – 5, 7, 10, 11 and 12, called delM(C1)) and four pups (animal numbers – 2, 3, 4 and 6 called delM(C2)) respectively. In delF out of 12 animals 4 animals formed 3 separate groups with 1 animal in 2 clusters (1 and 11, left from further analyses), 2 (animal number 5 and 6) in the 3rd cluster (delF(C2)). The remaining eight animals in the delF group were merged in to one cluster and was referred to as delF(C1). Single animals were left out as there were not enough data from single animals to do further analyses.

Using the above groups of animals, WTM, WTF, delM(C1), delM(C2), delF(C1) and delF(C2) we performed the same analysis of *KLD* between their respective distributions of syllable type occurrence (as in Figure 2). The *KLD* between each pair of the above groups are shown in Figure 8(B). The delF(C1) was found to be not different from the WTM and WTF, showing that the differences observed in the delF group in our analysis is only for the pups in delF(C2) or animal numbers 5 and 6 and left out animals 1 and 11 in the group. The majority 8/12 were like the WT groups. Further in the delM both C1 and C2 clusters were significantly different from the WT groups however delM(C1) was closer to the WT group. The animal numbers show that the animals of the same litter could belong to either cluster and hence the effect was not litter specific. Similarly in the delF group, delF(C2) – animal numbers 5 and 6, belonged to a litter whose animals were also present in delF(C1).

We extended the above analyses for joint distributions of starting 2 and starting 3 syllables of a bout. The results are summarized in Figure S7(A), (B) respectively for the two cases. The figures are arranged in the same way as Figure 8 with the dendrograms on the left and *KLD* between pairs of groups/clusters to the right. In each case the maximum distance for WTM needed to cluster the WTM male in one group was used as threshold for clustering. Based on these results as well, no litter specific effect was found. However both in the delM and delF groups we find some clusters very similar to the WTM and WTF showing that the alterations observed is not present in all delM pups. Similarly the majority of delF pups in all cases were similar to the WT groups, showing that only a small number of pups in delF group had altered sequence of vocalizations. However, it may be noted that in the delF animals, the animals with numbers 1, 5, 6 and 11 consistently in all three analyses (Figures 8 and S7) were different from the rest of the pups. It is because of these specific mice that the delF population showed differences in structure of sequences from the WT groups. The rest of the pups in the delF group together formed clusters and were same as the WT groups. However, such specific pups could not be found in the delM group showing large degree of heterogeneity in the sequence of vocalizations produced by the delM group.

## 4 DISCUSSION

Through our analyses of vocalization sequences we observe a primarily male specific alteration in vocalizations and vocalization sequences in the 16p11.2del mouse model of ASDs, while considering PICs emitted by WT control M and F groups and 16p11.2del M and F groups of pups. The primarily male specific alterations are present not only in basic call features like call rate, call durations and mean peak frequencies but also in considering syllable type distributions, joint distributions of pairs or triplets of syllables and further in length of dependence of syllables on previous syllables. The male and female WT pups were found not to differ in any of the above properties, while the sex specific differences in the 16p11.2del group was apparent in all the above properties. As shown with our clustering analyses, the differences could not be attributed to specific litters in the delM population. The departure observed in the delF group in the above properties could be attributed to only 4 particular pups of the 12 delF pups studied, while the rest were primarily like the WT populations. The most important result in the current study is the observation of the presence of dependence between in the syllable types in successive PICs in the WT, showing that PICs were not random sequences of syllable types but on average there was a great degree of dependence between successive syllables. Such dependence is completely absent in the delM group, which produce random syllable types in a sequence in general.

The current study is limited to PICs produced only at P5 and hence it is desirable to see if there were systematic changes in the developmental time course over age till the pups are weaned and then in context specific vocalizations produced in adulthood.^9^ Although a previous study^23^ had shown the non‐random nature of PICs using Zipf's statistic, our study using information theoretic techniques had clearly parsed out the actual dependence present in sequences of PICs in the WT. We also identified specific syllable sequences that contribute to some degree to the dependence observed in sequences of PICs. It needs to be seen if over time there are further developmental changes in the sequences produced and how they were altered or if they recover at later ages in the 16p11.2delM population. Thus our study further strengthens the possibility of using the 16p11.2del mouse as model for studying development of vocalizations or delayed onset of proper sequences. Since 16p11.2 deletion syndrome in humans is associated with improper speech and language development our results allow the possibility of using the mouse model in understanding the circuit and mechanistic basis for such impairment. Further the analysis tools developed to study structure in mouse pup vocalizations can be easily used to study deficits in other ASD mouse model PICs as well as in adult context specific vocalizations.

It should be noted that at P5, the mouse auditory system is not developed and the pups are essentially deaf. Thus our results also imply that not just the PIC vocalizations themselves^50^ the PIC structure or sequencing observed at P5 is innate and not learned. However, it remains to be seen if the vocalization sequences as studied here over age change and require auditory experience or not. Since there is a male specific deficit in reward learning^51^ in the 16p11.2del mice, the production of PIC sequences seeking the mother at later ages could also have deficits if the later vocalizations are learned. Moreover, it is known that there are deficits in the adult social interaction related vocalizations in the 16p11.2del mice.^34^ However future studies are required to investigate exactly which aspects of vocalization sequences over age have deficits and if they can be mechanistically tied to the deficits in reward learning.

Although other motor deficits were not evident in the delM or delF population, alterations in the vocalization production machinery is not known, which could cause some of the observed changes but likely not the changes in order of syllables observed. Moreover, since all types of syllables were present in all groups except in different proportions and different types of sequences, it is unlikely that motor deficits can explain our observations. Further investigations with a larger dataset needs to be performed by extending the classification scheme to 10 or more syllable types as done in other work^25^ or with other spectrographic analyses^23^, ^48^ to provide more insight into the deficits in vocalizations and sex specificity we observe. Similarly the lower body weight of 16p11.2del pups^34^ could be a factor in inducing the observed higher frequency of calls because of the smaller size of the vocal organ. However, higher frequency in the S type calls in the 16p11.2del mice does not alter our observations regarding sequences.

The production of sequences of successive syllables might be specific to the early neural circuitry involved in vocalization production (one example site could be Layer V neurons in the motor cortex,^52^ which could be disrupted in the 16p11.2delM. No study to the best of our knowledge has addressed the issue of production of sequences, or order of syllables produced. A recent study^53^ shows increased cortical excitation inhibition ratio as a common theme in ASDs, with four mouse models including the 16p11.2del. However, the study did not consider any sex‐specific effects. It also remains to be investigated if the ASD related excitation inhibition ratio change is present in structures involved in vocalization production. Imbalance of excitation and inhibition could be a possible mechanism by which sequences of vocalization syllables may be altered, however, a circuit model for the production of sequences needs to be tested.

A previous study^54^ shows specific changes in 16p11.2del mice with increased Layer VI cortico‐thalamic projection neurons and overall decrease in calretinin positive interneurons compared with WT. However, although changes were not observed in Layer V neurons, the nature of changes specific to motor cortex are not known. Furthermore, changes in interneurons of other types (e.g., parvalbumin, somatostatin) have not been studied in this particular mouse model. Such studies would allow framing hypotheses about the circuit level alterations that may mechanistically explain the observations in our study.

## CONFLICT OF INTEREST

The authors declare no conflicts of interest.

## Supporting information


**FIGURE S1** Probability of occurrence of each syllable type with 160 and 180 kHz as the upper cut‐off frequency. The figure shows the proportion of each syllable type for WTM, WTF, delM and delF using 180 kHz (gray) and 160 kHz (black) as the upper cut‐off frequency in pre‐processing. The distributions are exactly the same.

**FIGURE S2** Example spectrograms of types of syllables in each major category. The figure shows example spectrograms of syllables of each major type (Figure 1), which can be categorized further into different sub‐types. The different subtypes are identified in the subplot titles. The first row shows examples of noise (N) type syllables, the next three rows show no jump in pitch or S‐type and the next three rows are respectively examples of syllables with one pitch jump (J‐type), harmonics (H‐type) and other (O) type with two or more pitch jumps.

**FIGURE S3‐** Schematic of bout identification and calculation of significant *MI*. A) The silence duration between every successive syllable (*t*_*i*_) was compared to a threshold value (*T*), and if it was less then *T* it syllables were considered within a bout. When the silence duration exceeded *T* it marked the end of a bout and the subsequent syllable was the beginning of a new bout. Bouts in a sample sequence of tokens (color of the token identifies the syllable type) are marked with ellipses around them. B) Bouts were aligned together from the first position onwards for further analysis of dependence of syllables on the first syllable of a bout. C) *MI* between first the first syllable and syllables in the nth position was calculated based on the joint probabilities of the pair of syllables (syllable in first position and the *n* ^th^ position). D) In order to estimate the value of *MI* for no dependence case (ideally 0, but gives positive value for bias and limited data) comparisons were made with random scrambling of the order of syllables in every bout. *MI* was calculated for each random scrambling of order of all bouts to find the equivalent *MI* for no dependence case.

**FIGURE S4** Effect of varying threshold marking end of bout duration on *MI*. A) Bouts were identified as in Figure S3 with a particular value of threshold *T. T* was varied systematically *T* = mean(ISS) + k*std(ISS), ISS being the inter syllable silence distribution. *k* is varied from 1 to 3 in steps of 0.5. B) *MI* between first and nth syllable for varying *k*.

**FIGURE S5** Correlation among the joint distributions
Correlations between joint distributions of starting 2 syllables of bouts for each pair of joint distributions obtained by varying the threshold *T* marking end of bout (Figure S4) are shown.

**FIGURE S6** Schematic of calculating joint distributions
After identification of bouts (top row) bouts were aligned from the first position. In one case the first two syllables of a bout are considered (Figure 5A), shown by the vertically elongated ellipse in the depiction of syllables in bouts aligned by the first syllable (left, bottom row). In the other case any two successive syllables are considered (Figure 5B), shown by circles marking example pairs of syllables. From the pairs of syllables (middle columns, bottom row) of each case joint probability matrix was calculated based on the probability of occurrence of each possible pair type (bottom row right).

**FIGURE S7** Clustering of pups based on correlations of joint distributions. Both Figure S7A and B are arranged as Figure 8. In Figure S7A results of clustering are shown for joint distributions of first two syllables of bouts and Figure 6B shows the same for joint distributions of the first three syllables of bouts.

**FIGURE S8** Comparisons of call duration and mean peak frequency of different syllable types produced by the different groups of pups.

## Data Availability

All data in the manuscript are available on reasonable request.
