# Supplementary figures and images for: Male‐specific alterations in structure of isolation call sequences of mouse pups with 16p11.2 deletion
Source: Genes Brain Behav. 2020 Jul 6;19(7):e12681. doi: 10.1111/gbb.12681 (PMC7116069; doi:10.1111/gbb.12681)

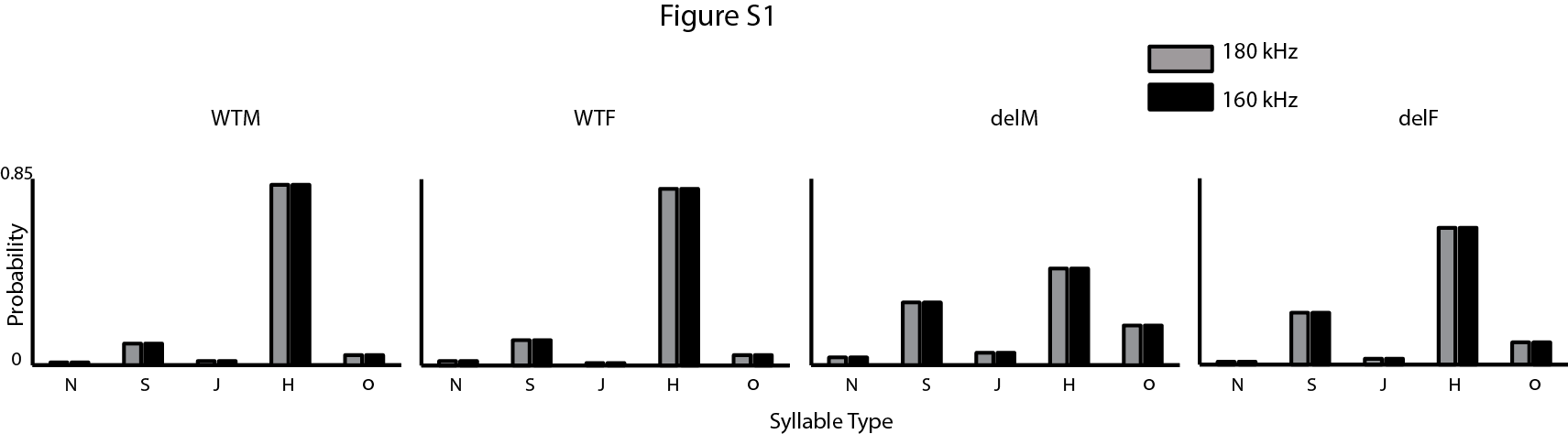

Supplement: Supplementary file 1 — FIGURE S1 Probability of occurrence of each syllable type with 160 and 180 kHz as the upper cut‐off frequency. The figure shows the proportion of each syllable type for WTM, WTF, delM and delF using 180 kHz (gray) and 160 kHz (black) as the upper cut‐off frequency in pre‐processing. The distributions are exactly the same. [file GBB-19-e12681-s001.zip › Figure_S1.png]

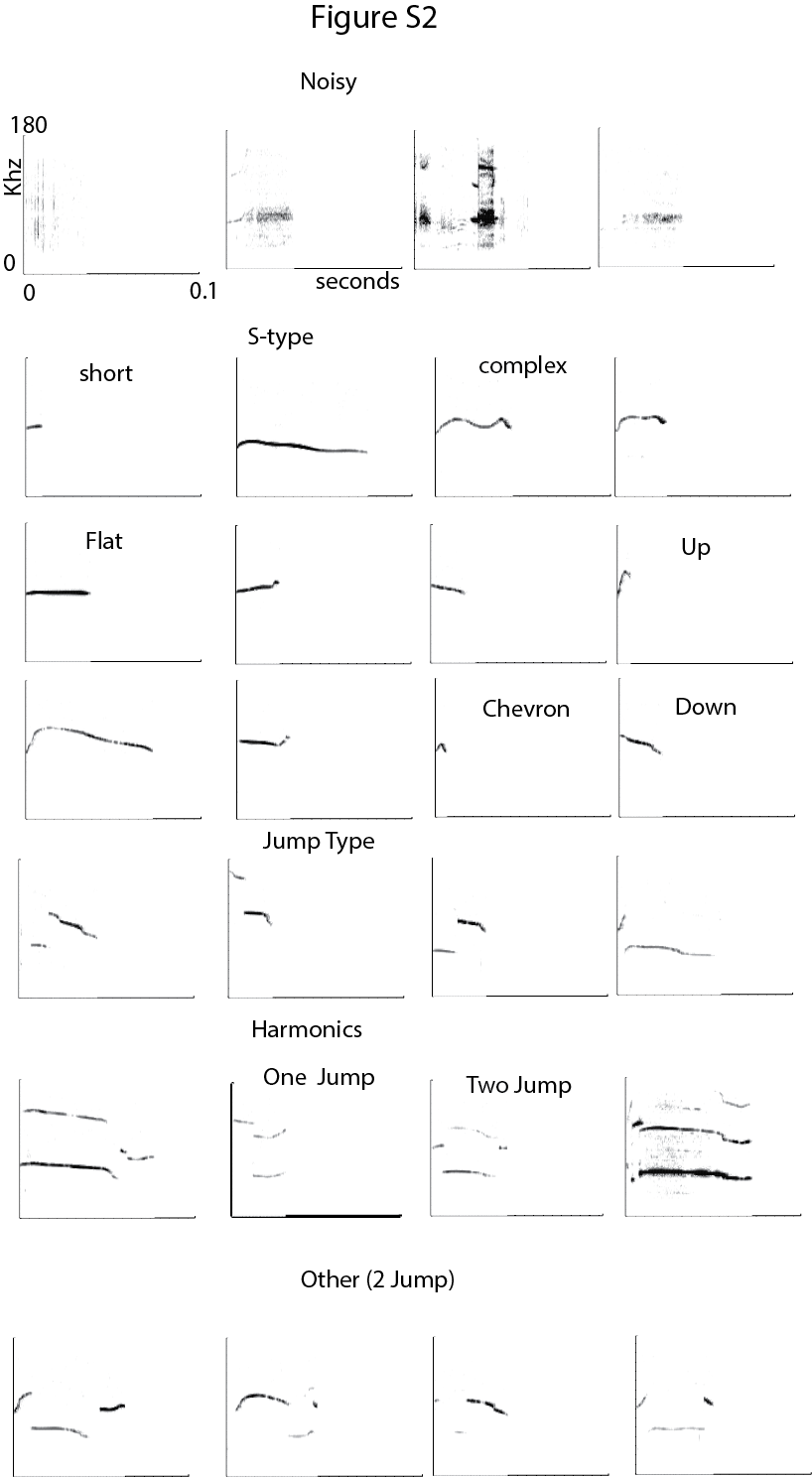

Supplement: Supplementary file 2 — FIGURE S2 Example spectrograms of types of syllables in each major category. The figure shows example spectrograms of syllables of each major type (Figure 1), which can be categorized further into different sub‐types. The different subtypes are identified in the subplot titles. The first row shows examples of noise (N) type syllables, the next three rows show no jump in pitch or S‐type and the next three rows are respectively examples of syllables with one pitch jump (J‐type), harmonics (H‐type) and other (O) type with two or more pitch jumps. [file GBB-19-e12681-s002.zip › Figure_S2.png]

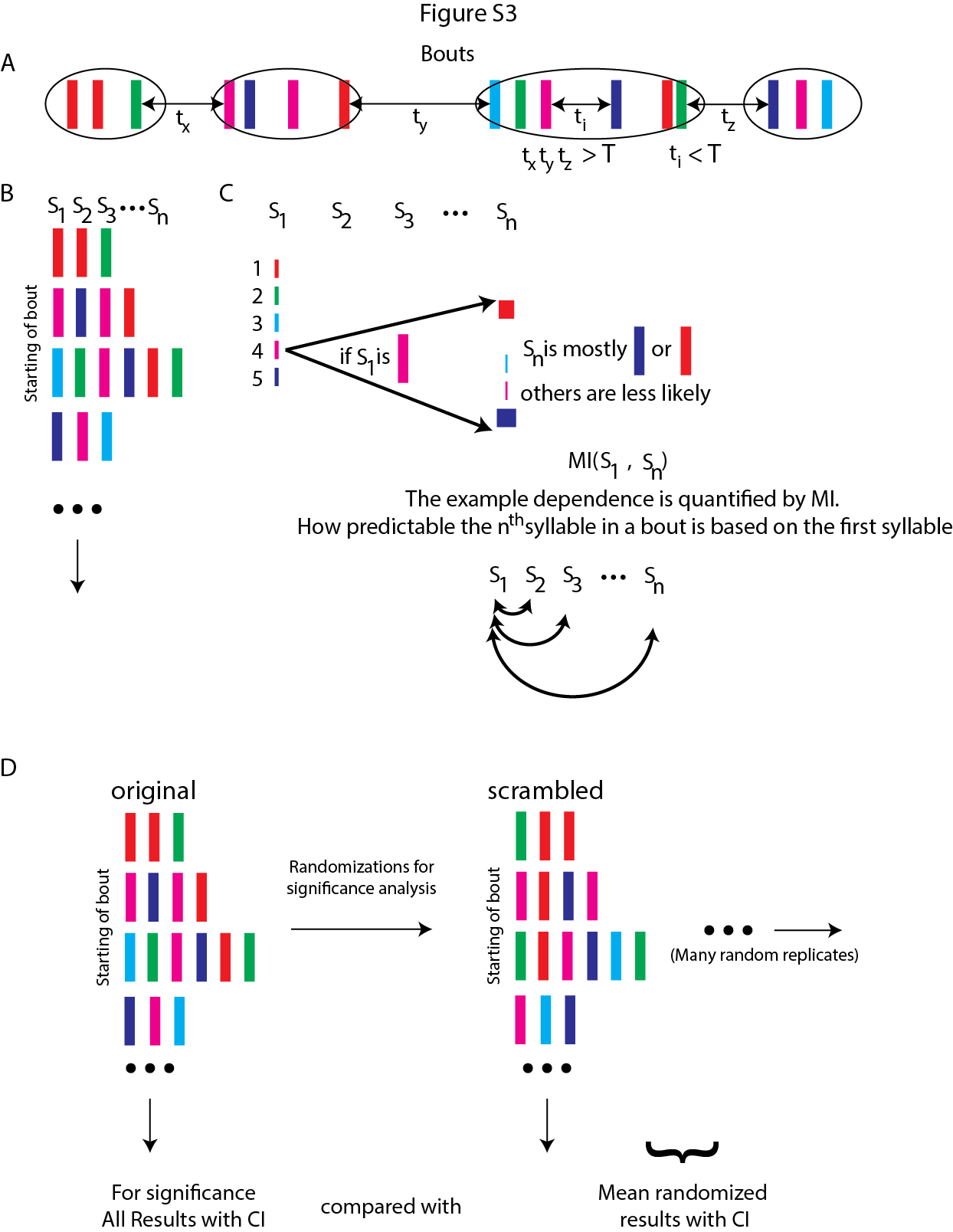

Supplement: Supplementary file 3 — FIGURE S3‐ Schematic of bout identification and calculation of significant MI. A) The silence duration between every successive syllable (t i) was compared to a threshold value (T), and if it was less then T it syllables were considered within a bout. When the silence duration exceeded T it marked the end of a bout and the subsequent syllable was the beginning of a new bout. Bouts in a sample sequence of tokens (color of the token identifies the syllable type) are marked with ellipses around them. B) Bouts were aligned together from the first position onwards for further analysis of dependence of syllables on the first syllable of a bout. C) MI between first the first syllable and syllables in the nth position was calculated based on the joint probabilities of the pair of syllables (syllable in first position and the n th position). D) In order to estimate the value of MI for no dependence case (ideally 0, but gives positive value for bias and limited data) comparisons were made with random scrambling of the order of syllables in every bout. MI was calculated for each random scrambling of order of all bouts to find the equivalent MI for no dependence case. [file GBB-19-e12681-s003.zip › Figure_S3.png]

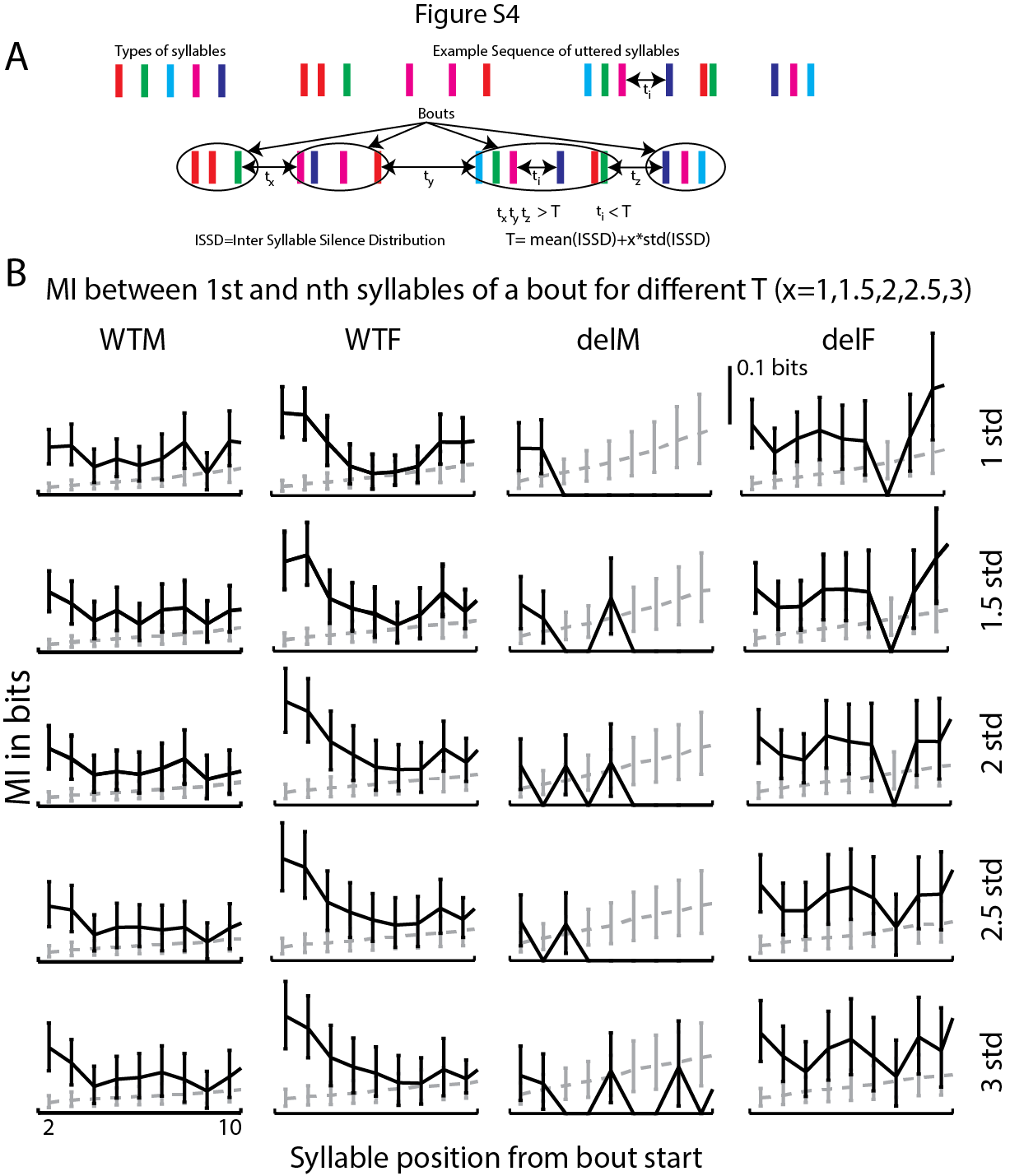

Supplement: Supplementary file 4 — FIGURE S4 Effect of varying threshold marking end of bout duration on MI. A) Bouts were identified as in Figure S3 with a particular value of threshold T. T was varied systematically T = mean(ISS) + k*std(ISS), ISS being the inter syllable silence distribution. k is varied from 1 to 3 in steps of 0.5. B) MI between first and nth syllable for varying k. [file GBB-19-e12681-s004.zip › Figure_S4.png]

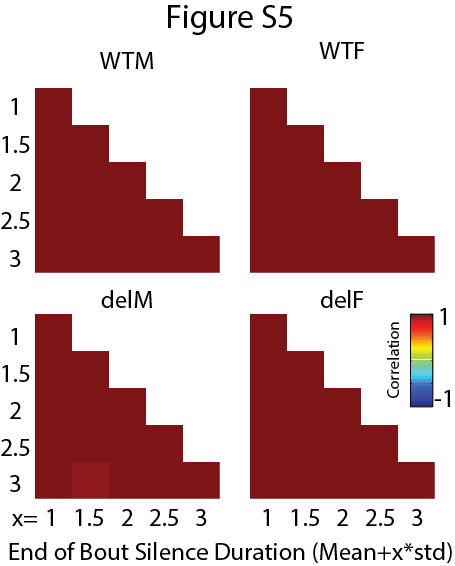

Supplement: Supplementary file 5 — FIGURE S5 Correlation among the joint distributions Correlations between joint distributions of starting 2 syllables of bouts for each pair of joint distributions obtained by varying the threshold T marking end of bout (Figure S4) are shown. [file GBB-19-e12681-s005.zip › Figure_S5.png]

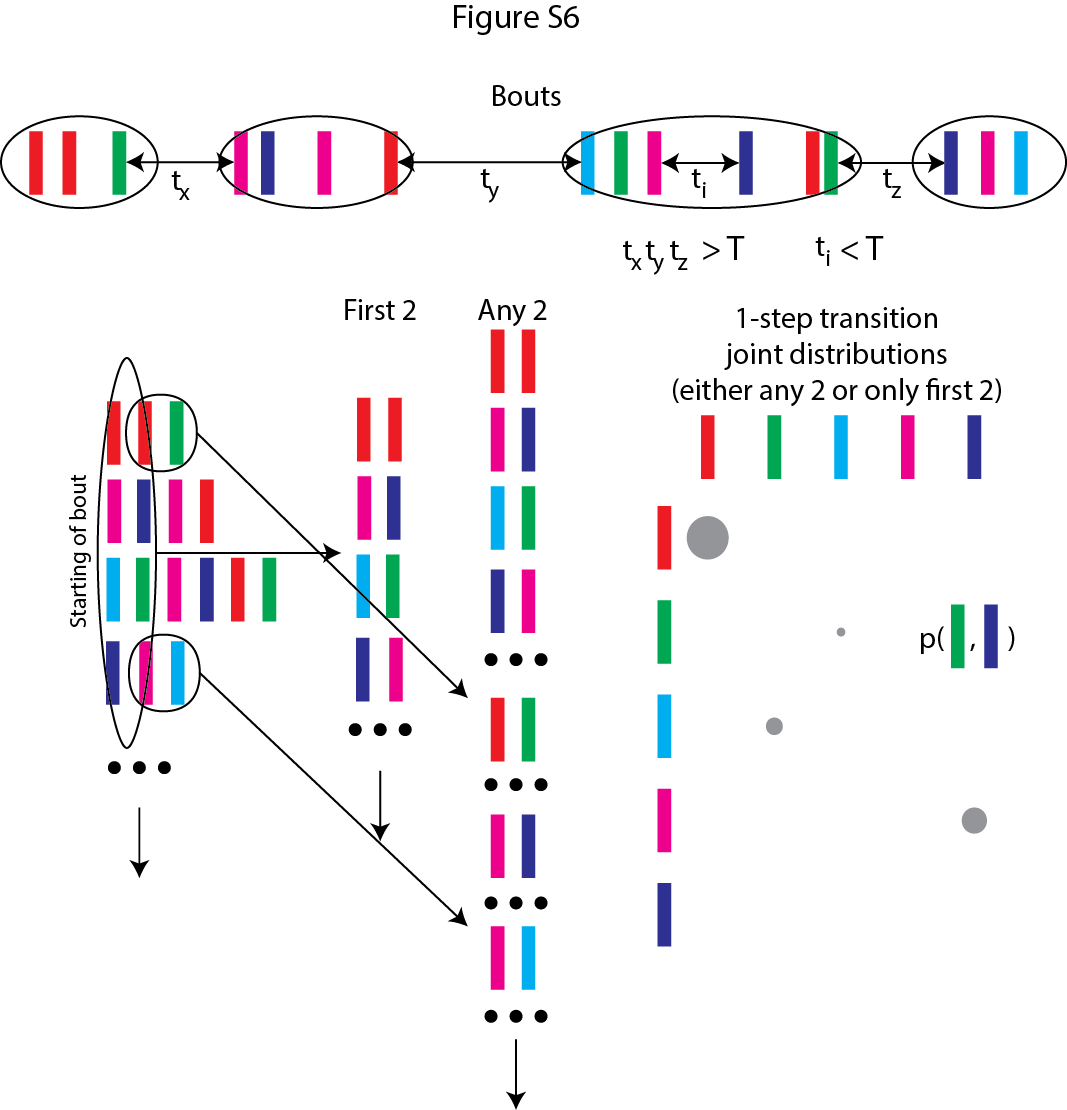

Supplement: Supplementary file 6 — FIGURE S6 Schematic of calculating joint distributions After identification of bouts (top row) bouts were aligned from the first position. In one case the first two syllables of a bout are considered (Figure 5A), shown by the vertically elongated ellipse in the depiction of syllables in bouts aligned by the first syllable (left, bottom row). In the other case any two successive syllables are considered (Figure 5B), shown by circles marking example pairs of syllables. From the pairs of syllables (middle columns, bottom row) of each case joint probability matrix was calculated based on the probability of occurrence of each possible pair type (bottom row right). [file GBB-19-e12681-s006.zip › Figure_S6.png]

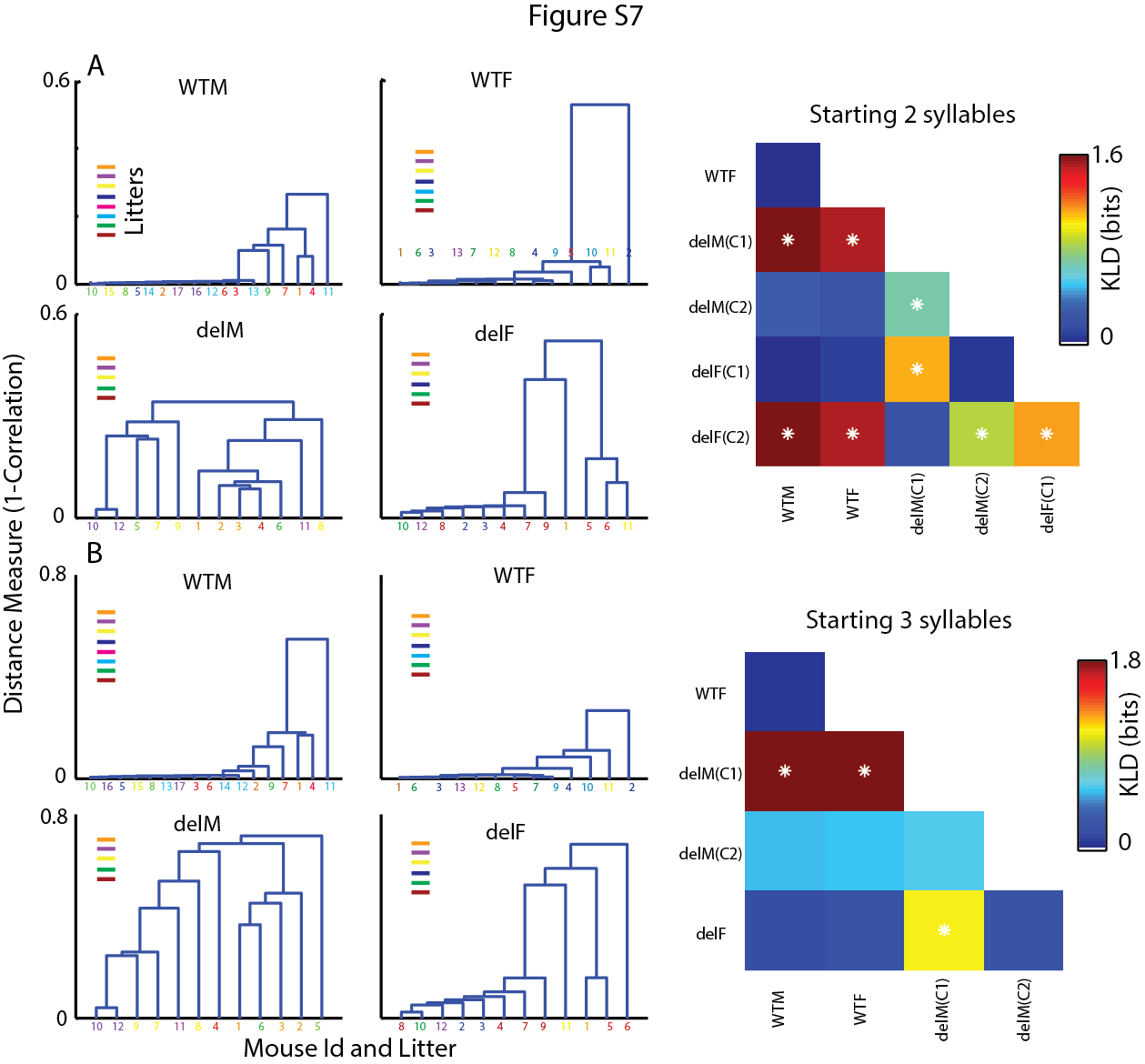

Supplement: Supplementary file 7 — FIGURE S7 Clustering of pups based on correlations of joint distributions. Both Figure S7A and B are arranged as Figure 8. In Figure S7A results of clustering are shown for joint distributions of first two syllables of bouts and Figure 6B shows the same for joint distributions of the first three syllables of bouts. [file GBB-19-e12681-s007.zip › Figure_S7.png]

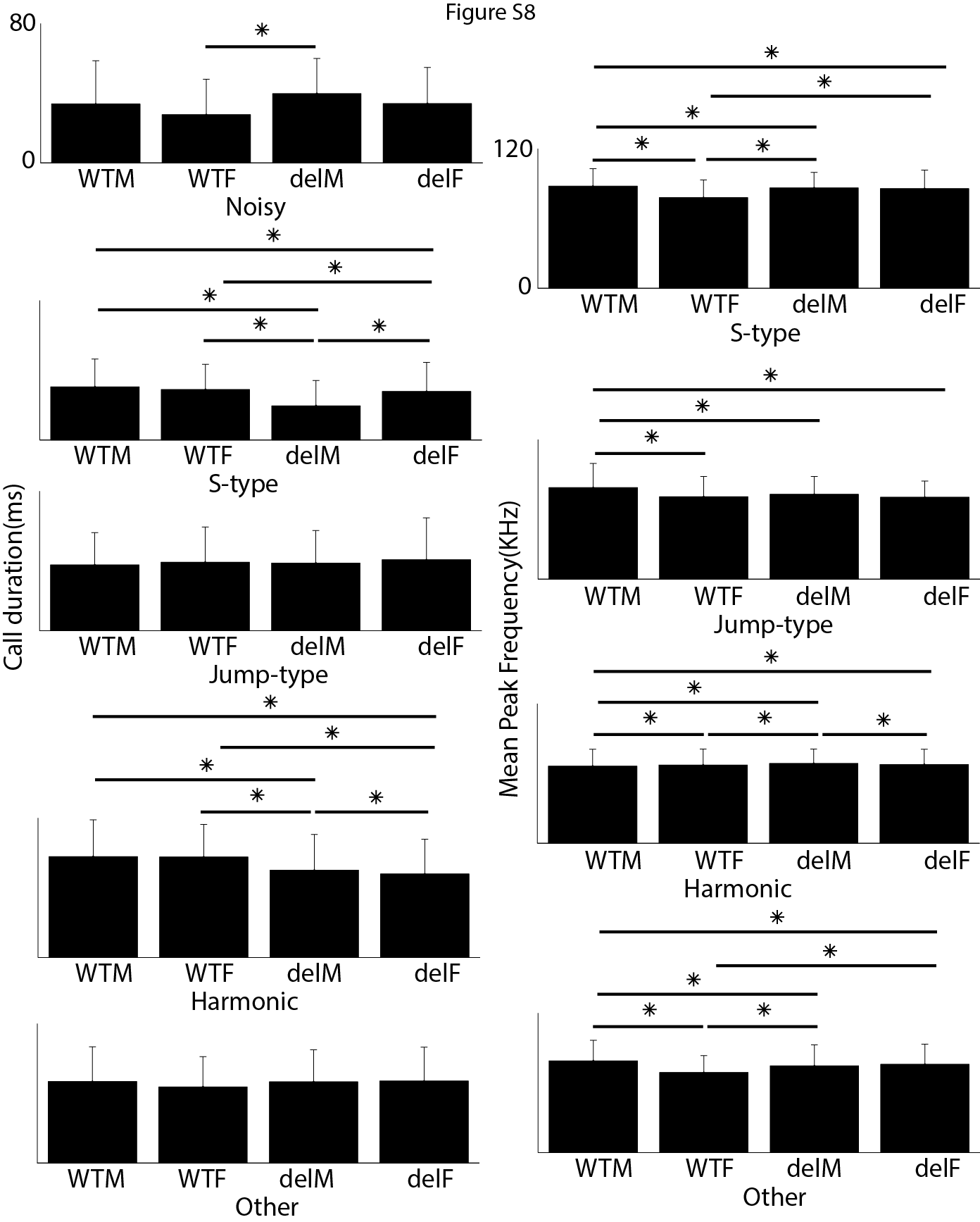

Supplement: Supplementary file 8 — FIGURE S8 Comparisons of call duration and mean peak frequency of different syllable types produced by the different groups of pups. [file GBB-19-e12681-s008.zip › Figure_S8.png]
